# Transmission Genetics of a *Sorghum bicolor* × *S. halepense* Backcross Populations

**DOI:** 10.3389/fpls.2020.00467

**Published:** 2020-04-30

**Authors:** Wenqian Kong, Pheonah Nabukalu, T. Stan Cox, Valorie H. Goff, Gary J. Pierce, Cornelia Lemke, Jon S. Robertson, Rosana Compton, Haibao Tang, Andrew H. Paterson

**Affiliations:** ^1^Plant Genome Mapping Laboratory, University of Georgia, Athens, GA, United States; ^2^The Land Institute, Salina, KS, United States; ^3^Center for Genomics and Biotechnology, Fujian Agriculture and Forestry University, Fuzhou, China

**Keywords:** genetic maps, autopolyploid, genotyping by sequencing, segregation, crop-to-weed

## Abstract

Despite a “ploidy barrier,” interspecific crosses to wild and/or cultivated sorghum (*Sorghum bicolor*, 2n = 2x = 20) may have aided the spread across six continents of *Sorghum halepense*, also exemplifying risks of “transgene escape” from crops that could make weeds more difficult to control. Genetic maps of two BC_1_F_1_ populations derived from crosses of *S. bicolor* (sorghum) and *S. halepense* with totals of 722 and 795 single nucleotide polymorphism (SNP) markers span 37 and 35 linkage groups, with 2–6 for each of the 10 basic sorghum chromosomes due to fragments covering different chromosomal portions or independent segregation from different *S. halepense* homologs. Segregation distortion favored *S. halepense* alleles on chromosomes 2 (1.06–4.68 Mb, near a fertility restoration gene), 7 (1.20–6.16 Mb), 8 (1.81–5.33 Mb, associated with gene conversion), and 9 (47.5–50.1 Mb); and *S. bicolor* alleles on chromosome 6 (0–40 Mb), which contains both a large heterochromatin block and the *Ma1* gene. Regions of the *S. halepense* genome that are recalcitrant to gene flow from sorghum might be exploited as part a multi-component system to reduce the likelihood of spread of transgenes or other modified genes. Its SNP profile suggests that chromosome segments from its respective progenitors *S. bicolor* and *Sorghum propinquum* have extensively recombined in *S. halepense*. This study reveals genomic regions that might discourage crop-to-weed gene escape, and provides a foundation for marker-trait association analysis to determine the genetic control of traits contributing to weediness, invasiveness, and perenniality of *S. halepense*.

## Introduction

Native to western Asia, *Sorghum halepense* L. (“Johnsongrass,” 2n = 4x = 40) finds occasional use as forage and even food (seed/flour), but is most noted as one of the world’s most noxious weeds, having spread across much of Asia, Africa, Europe, North and South America, and Australia and with the unusual distinction of being both a noxious weed and an invasive species (Quinn et al., 2013). Cytological, morphological, and molecular genetic data suggest that *S. halepense* is a naturally formed tetraploid hybrid derivative of *Sorghum bicolor* (2n = 20), an annual, polytypic African grass species which includes cultivated sorghum, and *Sorghum propinquum* (2n = 20), a perennial native of moist habitats in southeast Asia (Oyer et al., 1959; McWhorter, 1971; Paterson et al., 1995) estimated to have diverged from *S. bicolor* ∼1–2 million years ago.

The invasiveness of *S. halepense* is mainly owing to effective propagation by rapid flowering and disarticulation of mature inflorescences, together with underground rhizomes that can account for up to 70% of an individual plant’s dry weight (Oyer et al., 1959), store nutrients, and quickly produce new vegetative growth after quiescent periods (cold or drought). To date, no herbicide has been found to eradicate *S. halepense* without damaging sorghum—moreover, at least 24 herbicide-resistant *S. halepense* biotypes (Heap, 2012) are known.

Its ability to cross with cultivated sorghum (*S. bicolor*) makes *S. halepense* a paradigm for the dangers of crop “gene escape” (Dale, 1992; Ellstrand, 2001; Morrell et al., 2005), with engineered improvements of sorghum raising concerns about the potential to increase persistence and/or spread of this weedy and invasive plant (Arriola and Ellstrand, 1996; Tesso et al., 2008). Although differing in ploidy from *S. halepense*, *S. bicolor* can serve as the pollen parent of triploid or tetraploid hybrids (Warwick and Black, 1983; Hoang-Tang and Liang, 1988). The “Johnsongrass” of North America has been extensively affected by introgression from *S. bicolor* (Morrell et al., 2005) like *Sorghum almum*, commonly known as Columbus Grass (Warwick et al., 1984). Introgression from *S. bicolor* to *S. halepense* has persisted in non-random regions of the genome, associated with seed size, rhizomatousness, and levels of lutein, an antioxidant implicated in cold tolerance (Paterson et al., submitted).

From a different perspective, however, *S. halepense* harbors many characteristics that may increase agricultural productivity if transferred to sorghum (Sangduen and Hanna, 1984). It flowers and produces seeds rapidly, is resistant to many diseases and insects, and adapts to a wider range of environments than both of its progenitors. *S. halepense* might also contribute to breeding of genotypes suitable for multiple harvests from single plantings (Cox et al., 2002; Glover et al., 2010; Paterson et al., 2013).

Here, we report genetic maps of two BC_1_F_1_ populations derived from different tetraploid F_1_ progenies from a cross of *S. bicolor* BTx623 (recurrent parent) × *S. halepense* (Gypsum 9E) and reveal chromosomal characteristics and segregation patterns using genotyping by sequencing (GBS). In comparison to its progenitors *S. bicolor* and *S. propinquum*, the chromosomal composition of *S. halepense* sheds light on its evolution. Patterns of transmission of alleles from *S. bicolor* and *S. halepense* to interspecific progenies provide evidence of genomic regions that may, respectively, be favorable or recalcitrant to interspecific gene flow. This information identifies potential locations for transgenes or other genetic modifications (“edited” alleles) that may minimize crop-to-weed gene flow. These two populations are also of potential agronomic importance: identifying and incorporating novel alleles conferring yield potential, nitrogen fixation, insect or disease resistance, and rhizomatousness may benefit current or future sorghum breeding programs.

## Materials and Methods

### Genetic Stocks

Two tetraploid F_1_ hybrids (H4 and H6) derived from crossing tetraploid *S. bicolor* BTx623 (colchicine-induced) × *S. halepense* (G9E) (Cox et al., 2018) were backcrossed to a tetraploid version of the recurrent parent, *S. bicolor* BTx623: two BC_1_F_1_ mapping populations, of 146 H4-derived and 108 H6-derived individuals, respectively, were developed. BC_1_F_2_ rows derived from selfed seed of a single BC_1_F_1_ plant were planted at the University of Georgia Plant Science Farm, Watkinsville, GA, United States, on 28 May 2013 and 9 May 2014, and at The Land Institute, Salina, KS, United States, on 3 June 2013 and 17 June 2014. Plants were harvested for phenotyping when the main head reached senescence.

### Genotyping by Sequencing (GBS)

Leaf samples of the BC_1_F_1_ individuals were frozen at −80°C and lyophilized for 48 h. Genomic DNA was extracted from the lyophilized leaf samples based on Aljanabi et al. (1999). Genome sequencing was conducted in Fujian Agricultural and Forestry University (FAFU) genome sequencing center. The GBS platform used a slightly modified version of Multiplex Shotgun Genotyping (MSG) (Andolfatto et al., 2011) combined with the Tassel GBS5 v2 analysis pipeline (Glaubitz et al., 2014). Sequencing used an Illumina HiSeq 2500, Rapid V2 kit that generated about 150 million reads of 100 base pair (bp) fragments per run with single-end sequencing. The restriction enzymes H*in*p1I and *Hae*III were used in GBS to construct the library. Adapter sequences can be found in the Supplementary Material. The dsDNA concentration was measured (20 ng/μL) and normalized across 96 individuals before library construction. Libraries were PCR-amplified to enrich for adapter-ligated fragments. Size selection was performed at 250–300 bp using “QIAquick Gel Extraction Kit.”

### Genotype Calling and Filtering

Genotypes were determined by single nucleotide polymorphism (SNP) “calling” based on the reference genome of *S. bicolor* BTx623 *v1.4* (Paterson et al., 2009). Using Tassel-GBS 5 (Glaubitz et al., 2014), the first 90 bp of each read were mapped onto the reference genome. SNPs were “called” based on alignment of the reads to the reference genome. An in-house pipeline was used to determine the genotypes for these two populations, as follows:

1 Raw SNPs were first thinned out within 100 bp, since SNP sites close to each other or on the same read provide little non-redundant information in early generations following crossing.
2 Biallelic SNP markers with an average depth of 10 were selected.
3 The PL (phred-scaled genotype likelihoods) field from the raw VCF file consisted of three floating point log10-scaled likelihoods for AA, AB, and BB genotypes where A is the reference allele and B is the alternative allele (Danecek et al., 2011). The PL field was transformed into probability scales by 10^(–PL/10)^. Genotype calling used the field with the minimum PL value, except that a missing genotype was assigned if the second largest probability of a genotype is greater than 0.05 for each individual at each locus.
4 Homozygous genotypes with lower than 6x coverage were considered missing data.

A total of 2240 raw polymorphic markers were obtained after the *genotyping* and *filtering* steps described above for both H4- and H6-derived populations and used to analyze patterns of segregation.

### Map Construction

For each sorghum chromosome, we clustered markers based on a minimum LOD score of 10. Genetic distances were first estimated based on the physical orders of markers in the published sorghum genome (Paterson et al., 2009) and then markers within 1cM bins were combined. Bin genotypes were defined as follows: If there was only one marker in the bin, the bin genotype would be the same as the marker genotype; if there were more than one marker in the bin, bin genotypes would be determined by merging marker genotypes to minimize missing data points. Using the combined genotype file, *de novo* marker ordering was implemented for each corresponding sorghum chromosome and the final genetic map was constructed using R/qtl with the Kosambi mapping function. The map distance was calculated with an error probability of 0.01 (Broman et al., 2003). SNP marker co-ordinates to sorghum reference genome v3.1 are provided in Supplementary File S1.

### Analysis of Segregation

Using the R program (R Core Team, 2013), a chi-squared test was applied to each marker to test the hypothesis that it deviated significantly from a ratio of 5:1.

### Whole Genome Polymorphism Analysis

A total of four genotypes, *S. bicolor* IS3620C (SRX2158431), *S. propinquum* from University of Georgia (SRX030701 and SRX030703), *S. propinquum* from Australia (SRX208587 and SRX208588), and *S. halepense* (SRX142088), were included in whole-genome SNP analysis against the *S. bicolor* BTx623 v1.4 reference genome. The Burrows-Wheeler Aligner (BWA) MEM algorithm was used for read alignment (Li and Durbin, 2009). Variant calling used samtools/Bcftools (Li, 2011). Data were filtered with a minimum phred score of Q20, and a minimum depth of 10 with a maximum missing data of 30% for each SNP locus.

## Results

### Genetic Mapping and Patterns of Segregation

For three sets of 96 individuals, sequencing read depths of 360.7, 181.2, and 175.6 million yielded 689,684 raw SNP markers, which were thinned to 215,341 by removing loci within 100 bp of another locus. Of the 254 genotyped individuals, eight were deleted due to very low sequence coverage leaving 141 from the H4 population, and 105 from H6. After filtering steps (see section “Materials and Methods”), the same 2240 polymorphic markers with a minimum average depth of 10 at each locus were used for genetic mapping of each population.

**FIGURE 1:**
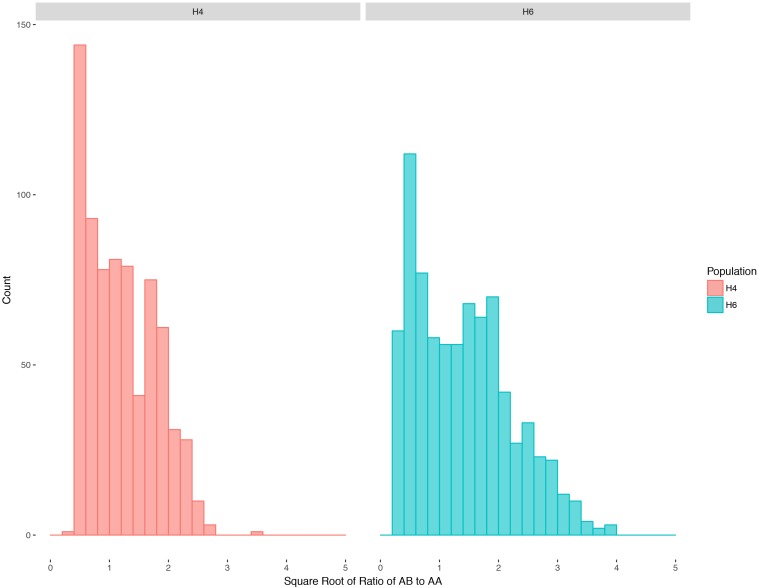
Distribution of segregation ratios (square root transformed) for H4 and H6 populations of *S. bicolor* BTx623 × *S. halepense* G9E with mapped markers. AA is the homozygous genotype while AB is the heterozygous genotype.

Ratios of heterozygotes to homozygotes for all mapped markers after square-root transformation (Figure 1) show a continuous distribution, indicating a mixture of disomic and polysomic inheritance as observed in other tetraploids (Jannoo et al., 2004; Stift et al., 2008). Autotetraploids can segregate in a variety of manners, including random chromosome segregation, random chromatid segregation, and maximum equational segregation, and can be further complicated by varying degrees of double reduction (Gupta, 2007). Random chromosome segregation assumes no crossing over between a gene and the centromere, while maximum equational segregation assumes that such crossing over always occurs. An intermediate state between random chromosome segregation and maximum equational segregation is often more frequent than the two extremes (Gupta, 2007). With random chromosome segregation (Muller, 1914), the expected segregation ratios for these populations are 1:1 (heterozygotes:homozygotes) for simplex markers and 5:1 for duplex markers. Under random chromatid segregation, where a chromatid can end up with any chromatid in a gamete with equal frequency, the segregation ratio can be 13:15 (simplex) or 11:3 (duplex) (Haldane, 1930). With maximum equational segregation (Mather, 1935), the segregation ratio can be 11:13 (simplex) or 7:2 (duplex).

**FIGURE 2:**
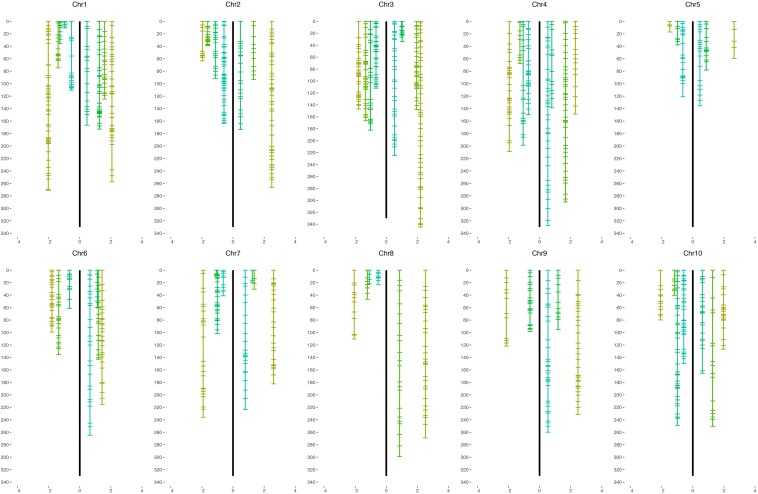
Genetic maps of the BC_1_F_1_ population of *S. bicolor* BTx623 × *S. halepense* G9E. The maps of the H4-derived population are on the left of the black line and H6 maps are on the right for each sorghum chromosome. The *x*-axis is the average segregation ratio of each linkage group after square-root transformation.

We grouped all 2240 selected SNP markers based on pair-wise recombination fractions using relatively stringent thresholds in R/qtl (Broman et al., 2003), mapping 722 and 795 to 38 and 36 linkage groups spanning 3896.5 and 6048.4 cM for the H4- and H6-derived populations, respectively (Tables 1, 2 and Figure 2). For individual sorghum chromosomes, we obtained two to six linkage groups, some covering only portions of the underlying chromosome (Figure 2) but others due to highly divergent segregation patterns of different allele groups (below).

**TABLE 1:** Characteristics of the genetic map of the H4-derived BC_1_F_1_ population of *S. bicolor* BTx623 × *S. halepense* G9E.

LG‡	Marker No.	Length (cM)	Avg spacing (cM)	Max spacing (cM)	Avg No. AA ‡	Avg No. AB	AB/AA ratio
1A	49	271.1	5.65	34.45	24.63	94.61	3.84
1B	16	74.8	4.98	18.95	42.94	74.31	1.73
1C	9	35.9	4.49	7.08	47.11	77.11	1.64
1D	5	10.8	2.71	3.14	56.40	52.00	0.92
1E	20	110.6	5.82	27.36	95.15	27.10	0.28
2A	11	63.4	6.34	32.51	23.55	89.55	3.80
2B	19	39.3	2.18	8.04	33.74	85.74	2.54
2C	22	91.7	4.37	14.18	52.32	64.41	1.23
2D	47	164.2	3.57	18.09	89.04	29.28	0.33
3A	31	146.8	4.89	27.67	28.94	88.65	3.06
3B	35	166.7	4.90	22.33	45.43	72.17	1.59
3C	22	182.3	8.68	42.99	60.59	60.18	0.99
3D	33	112.0	3.50	14.36	82.73	34.27	0.41
4A	29	208.8	7.46	24.88	25.62	89.93	3.51
4B	9	67.7	8.47	24.43	46.00	69.89	1.52
4C	18	198.8	11.69	56.32	56.56	57.50	1.02
4D	29	149.6	5.34	19.48	81.14	36.66	0.45
5A	4	17.1	5.71	9.65	39.75	86.25	2.17
5B	7	37.8	6.30	16.42	62.71	55.57	0.89
5C	21	121.0	6.05	21.07	87.19	33.67	0.39
6A	26	99.3	3.97	11.20	30.35	88.35	2.91
6B	23	135.4	6.16	21.14	44.26	75.13	1.70
6C	11	61.6	6.16	15.65	80.36	33.91	0.42
7A	24	236.0	10.26	38.84	27.04	90.29	3.34
7B	5	9.6	2.40	5.68	58.20	67.20	1.15
7C	25	101.6	4.23	9.91	58.60	56.68	0.97
7D	11	41.2	4.12	11.96	84.82	33.36	0.39
8A	12	110.5	10.05	26.00	24.50	92.50	3.78
8B	7	46.7	7.78	12.20	44.43	65.86	1.48
8C	4	22.0	7.33	8.39	53.50	63.00	1.18
8D	8	23.6	3.37	6.89	94.13	25.25	0.27
9A	13	121.7	10.14	32.60	20.92	92.15	4.40
9B	26	98.2	3.93	18.93	86.50	31.27	0.36
10A	15	79.9	5.71	24.93	24.20	92.80	3.83
10B	7	40.2	6.70	14.01	49.14	67.57	1.38
10C	35	248.9	7.32	18.81	62.91	59.29	0.94
10D	34	149.6	4.53	23.21	88.74	28.82	0.32

†LG: linkage group.

‡AA: homozygous genotypes matching *S. bicolor* BTx623; AB: heterozygous genotypes.

**TABLE 2:** Characteristics of the genetic map of the H6-derived BC_1_F_1_ population of *S. bicolor* BTx623 × *S. halepense* G9E.

LG‡	Marker No.	Length (cM)	Avg spacing (cM)	Max spacing (cM)	Avg No. AA ‡	Avg No. AB	AB/AA ratio
1A	38	257.5	6.96	40.72	17.97	68.63	3.82
1B	22	124.6	5.94	16.66	26.73	60.68	2.27
1C	31	172.9	5.76	21.01	34.23	50.77	1.48
1D	18	166.6	9.80	28.76	70.06	14.83	0.21
2A	48	266.3	5.67	18.33	12.90	73.10	5.67
2B	11	93.6	9.36	17.29	31.91	52.00	1.63
2C	22	173.5	8.26	32.11	67.45	14.82	0.22
3A	52	344.7	6.76	17.23	17.38	68.38	3.93
3B	30	147.8	5.10	15.71	19.37	65.63	3.39
3C	7	34.3	5.71	11.11	42.43	44.14	1.04
3D	5	26.6	6.65	9.12	45.00	42.80	0.95
3E	33	225.0	7.03	17.26	67.21	17.24	0.26
4A	15	148.7	10.62	19.71	15.33	66.47	4.33
4B	42	289.9	7.07	17.61	23.71	62.60	2.64
4C	13	138.7	11.56	33.11	51.62	29.92	0.58
4D	38	327.7	8.86	24.14	63.89	17.08	0.27
5A	5	59.6	14.89	18.80	11.00	72.20	6.56
5B	16	78.1	5.21	14.50	49.13	34.31	0.70
5C	19	135.6	7.53	22.71	76.16	15.21	0.20
6A	27	215.4	8.28	23.06	30.00	57.19	1.91
6B	17	142.9	8.93	19.86	38.24	47.59	1.24
6C	7	59.6	9.93	13.65	39.00	48.29	1.24
6D	27	264.9	10.19	27.41	60.56	22.22	0.37
7A	24	182.5	7.93	16.07	12.96	72.92	5.63
7B	4	30.5	10.18	13.25	34.25	57.00	1.66
7C	3	19.5	9.75	16.09	30.67	45.33	1.48
7D	26	223.5	8.94	20.07	54.58	25.50	0.47
8A	31	269.2	8.97	26.05	12.84	68.29	5.32
8B	22	299.0	14.24	29.05	48.23	29.50	0.61
9A	34	231.7	7.02	22.51	14.00	67.50	4.82
9B	13	95.5	7.96	16.56	35.77	47.15	1.32
9C	32	260.0	8.39	30.14	63.78	17.38	0.27
10A	20	126.7	6.67	19.38	21.35	67.10	3.14
10B	19	250.6	13.92	48.90	34.89	50.74	1.45
10C	24	165.3	7.19	36.34	65.00	21.29	0.33

†LG: linkage group.

‡AA: homozygous genotypes matching *S. bicolor* BTx623; AB: heterozygous genotypes.

### Transmission Genetics

Simplex markers should have segregation ratios of 1:1, 13:15, and 11:13, while duplex markers should have segregation ratios of 5:1, 11:3, and 7:2 for random chromosome segregation, random chromatid segregation, and maximum equational segregation, respectively. We lack the statistical power to distinguish with confidence among the three possible variations of simplex or duplex ratios, or intermediates. Therefore, we expect to find a total of four linkage groups for each sorghum chromosome including one *S. halepense* enriched group comprised of duplex alleles, two allele balanced groups with *S. halepense* simplex markers (one representing each *S. halepense* homolog in the F1 parent), and one *S. bicolor* enriched group comprised of duplex alleles. Since the *S. bicolor* parent is largely homozygous, linkage groups of *S. bicolor* simplex alleles are not expected. If chromosome pairing is disomic, the *S. bicolor* enriched group would be in repulsion phase with *S. halepense* groups, though the sample sizes of the two populations limit our ability to test this hypothesis.

We define linkage groups as either *S. halepense* or *S. bicolor* enriched based on statistically significant deviation from the expected segregation ratio of 1:1 for the average of all markers in the group. Linkage groups were deemed *S. halepense* enriched if the average segregation ratio of the entire linkage group is greater than 1.82 or *S. bicolor* enriched if it is smaller than 0.55 (calculated for 105 individuals by a Chi-squared test with 1 degree of freedom and an alpha value of 0.001), otherwise it is allele balanced (Table 3).

**TABLE 3:** Expected linkage groups and the thresholds.

Expected linkage groups	Possible marker type	Threshold
*S. halepense* enriched	Duplex	>1.82
Allele balanced	Simplex	>0.55 and =1.82
*S. bicolor* enriched	Duplex	=0.55

In the H4-derived population, six chromosomes (2, 3, 4, 7, 8, and 10) are largely congruent with the expected four linkage groups, although allele balanced groups are fragmented and do not provide full chromosome coverage of 4 and 8. Chromosome 1 is largely covered by both *S. halepense* and *S. bicolor* enriched groups, with three allele balanced fragmented linkage groups covering non-overlapping parts of the chromosome. Chromosome 2 has two *S. halepense* enriched groups and one *S. bicolor* enriched group but only one allele balanced group, with the less *S. halepense* enriched group (average 2.54 segregation ratio) possibly reflecting segregation distortion. For chromosomes 5 and 6, we only find one allele balanced linkage group, with both *S. halepense* enriched and one *S. bicolor* enriched (6) group only partly covering the chromosome(s). No linkage group segregating with an average ratio not significantly different from 1 was found on chromosome 9, perhaps suggesting a high density of duplex markers.

In the H6-derived population, chromosomes 6 and 7 have four linkage groups with the expected segregation ratios (albeit with incomplete chromosome coverage), while chromosomes 1 and 4 both have one *S. bicolor* enriched group but only one balanced group and two *S. halepense* enriched groups—this may reflect segregation distortion because both linkage group 1B and 4B have segregation ratios of 2.27 and 2.64. Chromosomes 2, 5, 9, and 10 each had only one allele balanced linkage group, while chromosome 8 had no allele balanced linkage groups. Chromosome 3 was particularly unusual, with a total of five linkage groups, deviating from our model in having two *S. halepense* enriched groups. However, the two allele balanced groups were extremely sparse, with questions about whether they truly overlap. If in fact they do not overlap, then segregation distortion could account for the extra *S. halepense* enriched group.

Detailed descriptions for each chromosome can be found in Supplementary File S1. Genetic maps can be found in Supplementary File S2.

### Segregation Distortion

**FIGURE 3:**
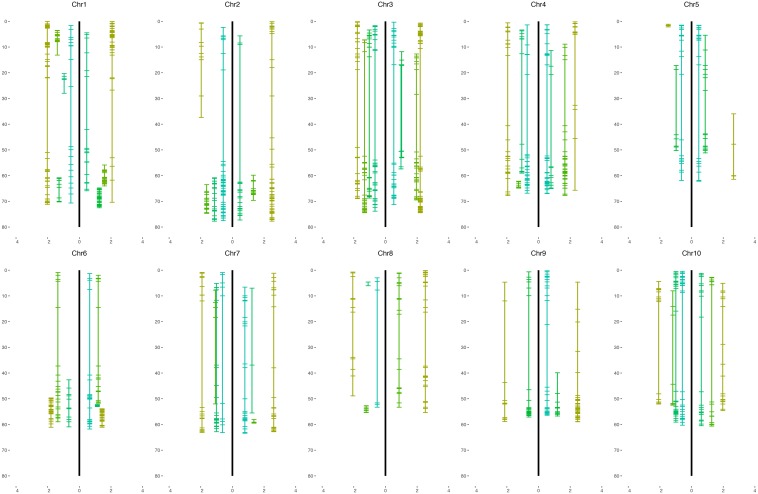
Physical coverage of the sorghum genome by each *S. bicolor* BTx623 × *S. halepense* G9E linkage group. The H4 population is on the left of the black line and the H6 population is on the right. The x-axis is the segregation ratio after square root transformation and that of the H4 population (left) is assigned a negative sign.

From the 2240 filtered markers, we detected totals of 53 and 80 SNP markers enriched for *S. halepense* alleles in the H4- and H6-derived populations, respectively, with heterozygote versus homozygote ratios significantly higher than 5:1 (*P* < 0.05, df = 1). Noting that these frequencies (53 and 80) are near the levels that could be expected by chance, further evidence was considered to discern whether some of these were true positives. The most compelling case for segregation distortion can be made for 22 markers significant at an alpha level of 0.05 in not just one but both populations. Finally, 57 markers are found significant from pooling the result of two populations (Supplementary File S3 and Figure 3). Regions on chromosomes 2 (1.06–4.68 Mb), 7 (1.20–6.16 Mb), 8 (1.81–5.33 Mb), and 9 (47.5–50.1 Mb) harbored at least three markers showing significant segregation distortion in each population. Interestingly, those regions completely lack markers segregating at 1:1 ratios, indicating aberrant transmission affected by selection or illegitimate recombination, hypotheses that warrant further investigation.

### Genomic Composition of *S. halepense*

Noting that *S. halepense* is a naturally occurring polyploid thought to derive from hybridization between *S. bicolor* and *S. propinquum*, to investigate its genomic composition we performed SNP “calling” with four genotypes: *S. bicolor* IS3620C, a race “guinea” accession that is highly diverged from BTx623 as a control; two *S. propinquum* accessions; and *S. halepense*. After filtering (see section “Materials and Methods”), we obtained a total of 8,703,936 SNP markers genome-wide with 1,777,782 (36.64%) identical to BTx623, 744,924 (15.35%) identical to *S. propinquum*, 447,479 (9.22%) heterozygous with one allele each from *S. bicolor* and *S. propinquum* (Table 4), 1,873,115 (38.61%) non-progenitor alleles, presumably arising from new mutation, and 3,852,257 unknown alleles due to missing data or polymorphism in *S. bicolor* (not included in calculating the percentages). A much smaller sample with SNP markers only from the genetic maps was also categorized into groups matching *S. bicolor*, *S. propinquum* alleles, and new mutations. For mapped SNP markers that were not polymorphic between the divergent *S. bicolor* races represented by BTx623 and IS3620C, a total of 36.72 and 42.41% of *S. halepense* loci retained *S. propinquum* alleles, while 52.48 and 46.56% are novel in the H4- and H6-derived populations, respectively (Table 4).

**TABLE 4:** Inferred SNP origins in the H4 and H6 populations of *S. bicolor* BTx623 × *S. halepense* G9E and in the whole genome.

SNP types	B^†^	H-BP^†^	H-PM^†^	N-M^†^	P^†^	Unknown^†^
Counts H4	7	79	1	423	296	300
(Proportion H4)	(0.87%)	(9.8%)	(0.12%)	(52.48%)	(36.72%)	–
Counts H6	5	71	1	325	296	296
(Proportion H6)	(0.72%)	(10.17%)	(0.14%)	(46.56%)	(42.41%)	–
Whole genome	1,777,782	447,479	8,379	1,873,115	744,924	3,852,257
(Proportion whole genome)	36.64%	9.22%	0.17%	38.61%	15.35%	–

*^†^B: alleles matching *S. bicolor* but not *S. propinquum*; H-BP: heterozygotes, matching both *S. bicolor* and *S. propinquum*; H-PM: heterozygotes, matching *S. propinquum* and a new allele; N-M: alleles not matching *S. bicolor* of *S. propinquum*, inferred to be new mutations; P: alleles matching *S. propinquum*, but not *S. bicolor*; Unknown: missing data from either *S. propinquum* or *S. halepense*, or polymorphism between S. *bicolor* BTx623 and IS3620C. Not included in calculating the percentages.*

The distribution of *S. halepense* alleles putatively derived from *S. bicolor* and *S. propinquum* indicates extensive recombination between progenitor chromosomes. There are only 26 (16 in H4, 19 in H6, and nine in both) non-random “runs” of 3 or more consecutive mapped loci with *S. propinquum* derived alleles, covering roughly 18.7% (H4) and 11.3% (H6) of the genome (Table 5).

**TABLE 5:** *S. halepense* G9E genomic regions with non-random “runs” of more than three consecutive *S. propinquum* alleles.

Chr	LGH4	LGH6	H4 start (Mb)	H4 end (Mb)	H4 range	H6 start (Mb)	H6 end (Mb)	H6 range
1	1A	1A	1,002,293	2,075,903	1,073,610	1,002,293	2,075,903	1,073,610
1	–	1A	–	–	–	4,118,965	5,421,700	1,302,735
1	1A	1A	9,156,903	9,747,197	590,294	9,156,903	9,747,197	590,294
1	1A	1A	10,172,236	11,113,096	940,860	10,172,236	11,113,096	940,860
1	1A	–	58,398,011	60,856,958	2,458,947	–	–	–
1	–	1B	–	–	–	60,795,489	61,898,805	1,103,316
1	–	1C	–	–	–	68,343,440	69,007,210	663,770
2	–	2A	–	–	–	37,375,688	49,746,108	12,370,420
2	2B	–	70,855,137	71,665,944	810,807	–	–	–
2	–	2A	–	–	–	72,907,102	73,093,086	185,984
3	3A	3A	7,974,628	10,505,659	2,531,031	7,974,628	10,505,659	2,531,031
3	–	3A	–	–	–	57,155,624	57,669,990	514,366
3	–	3A	–	–	–	66,256,314	66,512,680	256,366
3	3B	–	57,050,572	57,258,601	208,029	–	–	–
3	3B	3A	71,350,072	73,236,656	1,886,584	72,084,184	73,236,656	1,152,472
3		3E	–	–	–	5,529,567	7,162,059	1,632,492
3	3C	–	8,639,654	60,878,509	52,238,855	–	–	–
4	4A	4B	60,169,823	66,355,590	6,185,767	58,910,354	64,899,883	5,989,529
4	4B	–	64,423,184	64,876,657	453,473	–	–	–
4	4D		61,407,366	64,036,600	2,629,234	–	–	–
6	6A	6A	55,862,094	64,036,600	8,174,506	55,862,094	57,182,238	1,320,144
6	6A	6A	55,862,094	64,036,600	8,174,506	58,155,004	58,738,149	583,145
6	6B	6B	3,622,183	37,245,941	33,623,758	3,327,280	37,245,941	33,918,661
9	9A		57,335,220	58,856,483	1,521,263	–	–	–
9	–	9A	–	–	–	55,778,912	57,267,091	1,488,179
10		10B	–	–	–	52,116,222	59,317,021	7,200,799

## Discussion

Genetic maps of two BC_1_F_1_ populations derived from crossing of *S. bicolor* BTx623 and *S. halepense* G9E provide important new information about the genome-wide transmission genetics of crosses which may have aided the spread across six continents of *S. halepense* (“Johnsongrass”), and confer risks to “escape” of sorghum genes that could make *S. halepense* more difficult to control. Identification of DNA markers and construction of genetic maps will facilitate marker-trait association analysis and comparative studies with other sorghum populations. Revealing chromosomal characteristics, especially identifying non-random patterns of DNA marker distribution, provides information about underlying features of sorghum genome organization. An SNP profile sampling the breadth of the Sorghum genus illustrates the evolutionary path and fate of alleles from progenitors of *S. halepense*.

### GBS and Genetic Mapping in Polyploids

While GBS is a cost- and time-efficient method of finding SNP markers (Elshire et al., 2011; Poland et al., 2012), our coverage of each locus was not high enough to differentiate heterozygous genotypes with different dosages—nonetheless, we obtained adequate numbers of SNP markers to construct linkage maps in these two populations using allele presence/absence, and the unmapped markers may still be useful in analysis of marker-trait association.

For each of the basic sorghum chromosomes, we expect to find one linkage group segregating with a ratio of 5:1 (heterozygotes: homozygotes) derived from homozygous *S. halepense* loci, two linkage groups segregating with ratios of 1:1 from heterozygous loci on different *S. halepense* homologs, and one linkage group segregating with a ratio of 1:5 derived from homozygous *S. bicolor* loci.

**FIGURE 4:**
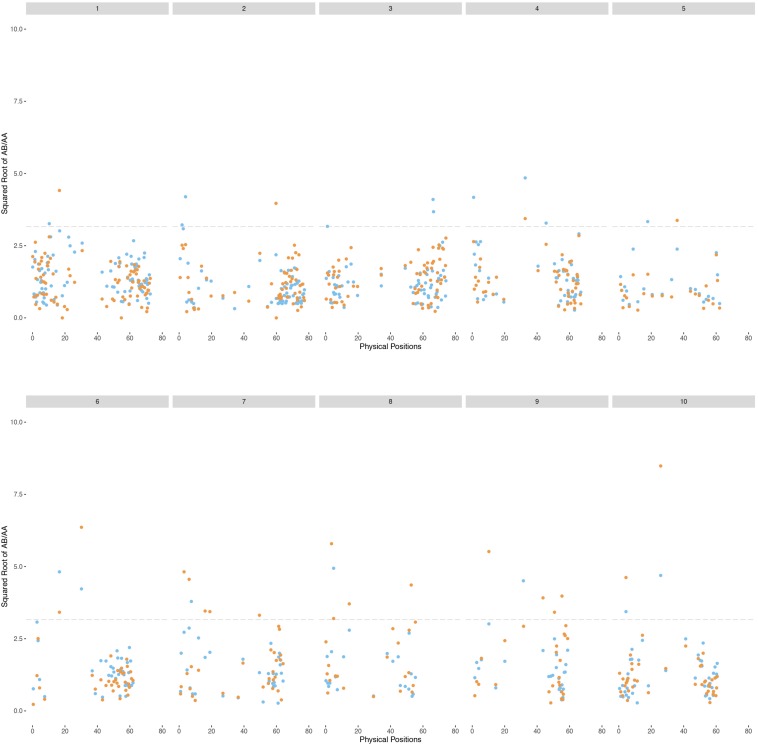
Patterns of segregation of the BC1F1 populations of *S. bicolor* BTx623 × *S. halepense* G9E based on the sorghum chromosomes. Square root transformation of the ratio of AB/AA for each marker are plotted in blue (H4-derived population) or orange (H6). AA is the homozygous genotype while AB is the heterozygous genotype.

Genetic maps of 722 and 795 loci comprising 38 and 36 linkage groups were generally congruent with the expected four linkage groups for each sorghum chromosome. However, noting that about 300 markers were necessary for the sorghum chromosomes to coalesce into largely complete linkage groups (Chittenden et al., 1994), it was not surprising to find incomplete chromosome coverage by some linkage groups. Marker distribution patterns of the H4 and H6-derived populations are generally similar (Figure 4), although varying somewhat in the number and segregation patterns of homologous chromosomes, suggesting differences in allele dosage. We consistently found at least one linkage group for each sorghum chromosome enriched with *S. halepense* alleles, segregating with ratios greater than 1.82 (heterozygotes: homozygotes), which is the upper 95% confidence limit for simplex markers segregating with a ratio of 1:1 (Table 1). We found one to three allele balanced linkage groups segregating with average ratios near 1 for most chromosomes; failure of finding two allele balanced linkage groups may be due to either fragmented pieces covering different portion of the chromosome, independent segregation from different homologous *S. halepense* chromosomes or not enough markers to coalesce the linkage group. In three cases (H4-2, H6-1, 4), segregation distortion along much of a linkage group appeared to shift otherwise allele-balanced groups into the *S. halepense* enriched category.

In principle, *S. halepense* enriched markers (segregating with ratios of approximately 5:1) and *S. bicolor* enriched markers (1:5) might comprise repulsion-phase associations of disomic alleles. To test this hypothesis, we reversed the genotyping of groups segregating with patterns of 1:5 and tried merging and ordering them together with groups segregating near 5:1. Such pairs of linkage groups either failed to coalesce or were only loosely connected to each other with relatively large genetic distances. Therefore, linkage groups segregating with average ratios of approximately 5:1 and 1:5 appear not to be in the repulsion state, although it remains possible that the sample sizes of the two populations are not large enough to detect linkage between some loci (Wu et al., 1992).

Chromosomes 5, 6, 8, and 9 are of particular interest, in that we fail to find linkage groups for certain ratios, or markers only cover parts of the corresponding sorghum chromosomes, suggesting aberrant chromosomal behavior caused by factors such as selection or preferential pairing. Only small portions of chromosomes 5 and 6 are covered by markers enriched with *S. halepense* alleles. A previous study (Bowers et al., 2003) of *S. bicolor* BTx623 × *S. propinquum* F2 population discovered a ribosomal DNA-enriched region with *S. propinquum*-dominated loci spanning 32.3–40 cM on chromosome 5, corresponding to 5–20 Mb in physical distance (Zhang et al., 2013). We find few *S. halepense* alleles in this region (Figure 4), possibly due to selection favoring an rDNA allele. Similarly, a large heterochromatin block on sorghum chromosome 6 is enriched for *S. bicolor* alleles. Chromosomes 8 and 9 each have a paucity of markers segregating with a ratio of 1:1. Further investigation is needed to understand these biases of marker distribution across the genome.

### Segregation Distortion

The overall distributions of segregation (Figure 1) in H4- and H6-derived populations suggest more intermediate than extreme segregation ratios (chromosome segregation and maximum equational segregation), consistent with other autopolyploids (Jannoo et al., 2004; Stift et al., 2008). Segregation distorted regions in these two populations may have causes including illegitimate recombination, unusual chromosomal events such as translocation and gene conversion (Wang et al., 2009), and gametic or zygotic selection. Fitness of progeny associated with particular alleles is being further investigated by QTL mapping. Regions of the *S. halepense* genome under strong selection may provide relatively “safe landing sites” for transgenes, i.e., with strong selection for *S. halepense* alleles reducing crop-to-weed gene flow from cultivated sorghum (Arriola and Ellstrand, 1996). With many different segregation patterns occurring in these populations, testing for segregation distorted regions requires stringent measures to avoid false positives. Nonetheless, four regions, on chromosomes 2 (1.06–4.68 Mb), 7 (1.20–6.16 Mb), 8 (1.81–5.33 Mb), and 9 (47.5–50.1 Mb), consistently have more *S. halepense* alleles than expected, and one region on chromosome 6 (0–40 Mb) has fewer than expected (Supplementary File S3).

Markers displaying segregation distortion might be linked to genes affecting fitness, for example, controlling fertility. To date, three sorghum genes controlling fertility have been located (Klein et al., 2005; Jordan et al., 2010, 2011), all proposed to encode pentatricopeptide repeat (PPR) proteins that are essential in the post-transcriptional process (Schmitz-Linneweber and Small, 2008). The interval on chromosome 2 (1.06–4.68 Mb) enriched for *S. halepense* alleles might be associated with *Rf2*, which is within the region from 5.4 to 5.7 Mb (Jordan et al., 2010). Similarly, the chromosome 8 interval (43.98–55.35 Mb in H6) enriched for *S. halepense* alleles in the H6 population harbors *Rf1*, based on flanking SSR markers Xtxp18-Xtxp250 located from 50.5 to 51.0 Mb. Segregation distortion on the short arm of chromosome 8 (1.81–5.33 Mb) overlaps with a region that has experienced frequent gene conversion (0.94–2.8 Mb), a mechanism that may cause segregation distortion (Wang et al., 2009, 2011).

The risk of “gene escape” into *S. halepense* constrains improvement of sorghum through biotechnology—many substantial benefits that could be realized by commercial use of transgenic *S. bicolor* are sacrificed due to risk of transgene escape into Johnsongrass, which has spread across more of the United States than sorghum is cultivated in, and continues to spread.

An attractive method for containment that is potentially effective and has minimal risk of public opposition is the targeting of transgenes to genomic regions recalcitrant to gene flow from sorghum. We identify several such candidate regions here, albeit based only on segregation in two populations derived from a cross between a single *S. halepense* genotype and an *S. bicolor* elite inbred, in a single environment. Clearly, the use of such regions for gene containment will depend first upon validating that the observed segregation distortions are reproduced in a broad sampling of genotypes and environments. Further, targeting of transgenes will require greater clarity as to the physical bounds of the genomic region that is recalcitrant to gene flow—such information might be obtained from fine-scale study either of large segregating populations or of large numbers of diverse accessions collected across the United States (for example), to precisely determine the loci responsible for segregation distortion in these regions. The co-evolution of its *S. bicolor*- and *S. propinquum*-derived subgenomes to adapt to cohabitation of a common nucleus in polyploid *S. halepense* may have resulted in many small chromosomal regions in which introgression from one ancestor may reduce fitness.

### Evolution of *S. halepense*

The *S. halepense* chromosomes consist of largely random distributions of *S. bicolor*-derived, *S. propinquum*-derived, and novel alleles, which indicates extensive recombination between *S. bicolor* and *S. propinquum*-derived “subgenomes.” It has been controversial whether *S. halepense* is an allo- or auto-tetraploid (Endrizzi, 1957; de Wet, 1978; Hoang-Tang and Liang, 1988; Fernandez et al., 2013). Since progenies of *S. bicolor* × *S. propinquum* crosses are fertile and show near-normal recombination, our previous studies (Paterson et al., 1995; Kong et al., 2013) have favored that *S. halepense* was auto-tetraploid. Comparing segregation patterns among two mapping populations and SNP distributions across the entire genome each further support the hypothesis that *S. halepense* is an autotetraploid, with its chromosomes a mosaic of alleles from *S. bicolor*, *S. propinquum*, and novel mutations (Table 4). Nevertheless, we found a total of 26 regions of the genome in either H4- or H6-derived population with non-random distribution of consecutive *S. propinquum* alleles in both populations (Table 5), including a total of eight regions occurring in both populations, on chromosomes 1 (3 regions), 3 (2), 4 (1), and 6 (2).

## Data Availability Statement

The raw data supporting the conclusions of this article will be made available by the authors, without undue reservation, to any qualified researcher.

## Author Contributions

WK performed the experiment, conducted the analysis, and drafted the manuscript. PN and TC developed the populations, collected phenotypic data, and conducted the analysis. VG, GP, CL, JR, and RC collected phenotypic and genotypic data. HT performed GBS sequencing. AP supervised the experiment and drafted and revised the manuscript.

## Conflict of Interest

The authors declare that the research was conducted in the absence of any commercial or financial relationships that could be construed as a potential conflict of interest.

## Supplementary Material

TABLE S1 Loci with non-Mendelian segregation combined for two BC_1_F_1_ populations of *S. bicolor* BTx623 × *S. halepense* G9E.

FILE S1 Details for each chromosome.

FILE S2 Details of genetic maps for the *S. halepense*-derived H4 and H6 populations.

FILE S3 Loci with non-Mendelian segregation combined for two BC_1_F_1_ populations of *S. bicolor* BTx623 × *S. halepense* G9E.
