# Supplementary material for: Transmission Genetics of a Sorghum bicolor × S. halepense Backcross Populations
Source: Front Plant Sci. 2020 Apr 30;11:467. doi: 10.3389/fpls.2020.00467 (PMC7203413; doi:10.3389/fpls.2020.00467)
Supplement: TABLE S1 — Loci with non-Mendelian segregation combined for two BC1F1 populations of S. bicolor BTx623 × S. halepense G9E. [file Table_1.docx]

Supplementary Table 1: Loci with non-Mendelian segregation combined for two BC_1_F_1_ populations of *S. bicolor* BTx623 × *S. halepense* G9E

|  |  |  | H4 |  | H6 |  |  | Pooled |  |
| --- | --- | --- | --- | --- | --- | --- | --- | --- | --- |
| SNP | Chr | Physical_dist | AB/AA | Sig | AB/AA | Sig | Both | AB/AA | Sig |
| S1_10311755 | 1 | 10.31 | 10.67 | * | 7.88 | NS | N | 9.35 | * |
| S1_16645888 | 1 | 16.65 | 9.11 | NS | 19.5 | ** | N | 12.3 | ** |
| S1_40908384 | 1 | 40.91 | 36 | *** | 15.6 | ** | Y | 23.3 | *** |
| S1_46405932 | 1 | 46.41 | 9.33 | * | 2.54 | ** | N | 4.81 | NS |
| S1_49998086 | 1 | 50 | 9.5 | * | 2.8 | * | N | 4.97 | NS |
| S1_55269769 | 1 | 55.27 | 24 | *** | 12.17 | * | Y | 16.9 | *** |
| S1_61787869 | 1 | 61.79 | 1.66 | NS | 11.14 | * | N | 3.04 | NS |
| S1_70587720 | 1 | 70.59 | 8 | NS | 11.5 | * | N | 9.31 | * |
| S2_1057029 | 2 | 1.06 | 8.67 | NS | 10.63 | * | N | 9.45 | ** |
| S2_1810453 | 2 | 1.81 | 10.38 | * | 6.33 | NS | N | 8.24 | * |
| S2_1826192 | 2 | 1.83 | 10.91 | * | 10.38 | * | Y | 10.7 | ** |
| S2_2112837 | 2 | 2.11 | 12.25 | * | 8 | NS | N | 10 | ** |
| S2_2592696 | 2 | 2.59 | 9.55 | * | 5.77 | NS | N | 7.5 | NS |
| S2_3982139 | 2 | 3.98 | 17.6 | ** | 6.44 | NS | N | 10.4 | ** |
| S2_4685750 | 2 | 4.69 | 18.67 | *** | 6.08 | NS | N | 10.3 | ** |
| S2_26594145 | 2 | 26.59 | 24.25 | *** | 26 | ** | Y | 25 | *** |
| S2_59755853 | 2 | 59.76 | 4.8 | NS | 15.75 | * | N | 6.63 | NS |
| S2_63532297 | 2 | 63.53 | 2.22 | NS | 36.5 | ** | N | 4.24 | NS |
| S2_66082501 | 2 | 66.08 | 3.06 | NS | 10.56 | * | N | 4.63 | NS |
| S2_67318879 | 2 | 67.32 | 2.76 | NS | 10.75 | * | N | 4.32 | NS |
| S2_70855137 | 2 | 70.86 | 3.56 | NS | 21 | ** | N | 6.05 | NS |
| S2_74686312 | 2 | 74.69 | 2.74 | NS | 10.75 | * | N | 4.26 | NS |
| S2_74899873 | 2 | 74.9 | 8.36 | NS | 10 | * | N | 9 | ** |
| S2_76168371 | 2 | 76.17 | 12 | * | 6.75 | NS | N | 9.2 | * |
| S2_76789433 | 2 | 76.79 | 2.92 | NS | 15.5 | * | N | 4.66 | NS |
| S3_700571 | 3 | 0.7 | 10.18 | * | 2.71 | ** | N | 5.06 | NS |
| S3_1062926 | 3 | 1.06 | 10.08 | * | 2.43 | ** | N | 4.73 | NS |
| S3_1135026 | 3 | 1.14 | 23.2 | *** | 27 | ** | Y | 24.6 | *** |
| S3_6722016 | 3 | 6.72 | 5.8 | NS | 33.5 | ** | N | 9.06 | * |
| S3_20644695 | 3 | 20.64 | 13.38 | ** | 19.5 | ** | Y | 15.4 | *** |
| S3_34250295 | 3 | 34.25 | 31 | *** | 21.25 | ** | Y | 26.1 | *** |
| S3_34250708 | 3 | 34.25 | 26.5 | *** | - | *** | N | 44.8 | *** |
| S3_57669990 | 3 | 57.67 | 1.54 | NS | 14.6 | * | N | 3.18 | NS |
| S3_66256314 | 3 | 66.26 | 16.86 | *** | 6 | NS | N | 9.62 | ** |
| S3_66505027 | 3 | 66.51 | 13.57 | ** | 3.82 | NS | N | 6.67 | NS |
| S3_66508407 | 3 | 66.51 | 10.8 | * | 7.55 | NS | N | 9.1 | ** |
| S4_805881 | 4 | 0.81 | 17.4 | ** | 7 | NS | N | 11 | ** |
| S4_806010 | 4 | 0.81 | 11 | * | 4.53 | NS | N | 7.12 | NS |
| S4_6311035 | 4 | 6.31 | 14.29 | ** | 17.75 | ** | Y | 15.5 | *** |
| S4_15434708 | 4 | 15.43 | 14.63 | ** | 17 | ** | Y | 15.5 | *** |
| S4_32653887 | 4 | 32.65 | 23.5 | *** | 11.83 | * | Y | 16.5 | *** |
| S4_45556066 | 4 | 45.56 | 10.8 | * | 6.5 | NS | N | 8.65 | * |
| S4_50435891 | 4 | 50.44 | 10.5 | * | 3.33 | NS | N | 5.83 | NS |
| S4_57722969 | 4 | 57.72 | 13.63 | ** | 28 | *** | Y | 17.5 | *** |
| S5_17017428 | 5 | 17.02 | 22 | *** | 3.48 | NS | N | 7.31 | NS |
| S5_17837434 | 5 | 17.84 | 12.1 | ** | 2.17 | *** | N | 4.65 | NS |
| S5_17839323 | 5 | 17.84 | 11.13 | * | 2.29 | ** | N | 4.72 | NS |
| S5_20824045 | 5 | 20.82 | 25.75 | *** | 17.25 | * | Y | 21.5 | *** |
| S5_35996091 | 5 | 36 | 5.67 | NS | 11.43 | * | N | 7.28 | NS |
| S5_61639044 | 5 | 61.64 | 1.93 | NS | 14.83 | ** | N | 3.51 | NS |
| S6_5884544 | 6 | 5.88 | 8.3 | NS | 11.83 | * | N | 9.63 | * |
| S6_16623205 | 6 | 16.62 | 23.25 | *** | 11.67 | * | Y | 16.3 | *** |
| S6_25520097 | 6 | 25.52 | 24.75 | *** | 24 | ** | Y | 24.4 | *** |
| S6_43674309 | 6 | 43.67 | 3.26 | NS | 11.43 | * | N | 4.94 | NS |
| S7_915336 | 7 | 0.92 | 10 | * | 1.41 | *** | N | 3.51 | NS |
| S7_1198601 | 7 | 1.2 | 10.88 | * | 24.67 | ** | Y | 14.6 | *** |
| S7_1624694 | 7 | 1.62 | 10.09 | * | 8.1 | NS | N | 9.14 | ** |
| S7_2805740 | 7 | 2.81 | 4.53 | NS | 13 | * | N | 6.56 | NS |
| S7_3048171 | 7 | 3.05 | 7.44 | NS | 23.25 | *** | N | 10.6 | ** |
| S7_3612606 | 7 | 3.61 | 4.24 | NS | 17.6 | ** | N | 6.47 | NS |
| S7_6161515 | 7 | 6.16 | 8.23 | NS | 20.75 | ** | N | 11.2 | ** |
| S7_6604014 | 7 | 6.6 | 3.88 | NS | 13.83 | * | N | 5.87 | NS |
| S7_6735921 | 7 | 6.74 | 1.73 | NS | 15 | * | N | 3.2 | NS |
| S7_6858502 | 7 | 6.86 | 1.4 | NS | 11.43 | * | N | 2.67 | NS |
| S7_7548864 | 7 | 7.55 | 14.38 | ** | 2.36 | ** | N | 5.27 | NS |
| S7_14222884 | 7 | 14.22 | 4 | NS | 14.6 | * | N | 6.04 | NS |
| S7_16072821 | 7 | 16.07 | 3.46 | NS | 12 | * | N | 5.27 | NS |
| S7_17895235 | 7 | 17.9 | 8.2 | NS | 12.8 | * | N | 9.73 | * |
| S7_18949389 | 7 | 18.95 | 4.13 | NS | 11.86 | * | N | 5.87 | NS |
| S7_37697004 | 7 | 37.7 | 4.48 | NS | 18.2 | ** | N | 6.93 | NS |
| S7_37954160 | 7 | 37.95 | 3.48 | NS | 10.71 | * | N | 5.17 | NS |
| S7_56916581 | 7 | 56.92 | 16.71 | *** | 8.33 | NS | N | 12 | *** |
| S7_57890358 | 7 | 57.89 | 1.51 | NS | 19.4 | ** | N | 3 | NS |
| S8_1813552 | 8 | 1.81 | 7.57 | NS | 11.14 | * | N | 8.76 | * |
| S8_3566394 | 8 | 3.57 | 4.21 | NS | 33.5 | ** | N | 7 | NS |
| S8_4847529 | 8 | 4.85 | 24.4 | *** | 10.25 | * | Y | 15.7 | *** |
| S8_5330663 | 8 | 5.33 | 5.93 | NS | 20 | * | N | 8.41 | * |
| S8_11551047 | 8 | 11.55 | 9 | NS | 4.67 | NS | N | 6.59 | NS |
| S8_14556229 | 8 | 14.56 | 7.83 | NS | 13.8 | * | N | 9.59 | ** |
| S8_41273697 | 8 | 41.27 | 10.11 | * | 8.71 | NS | N | 9.5 | * |
| S8_43980562 | 8 | 43.98 | 7.87 | NS | 13.14 | * | N | 9.55 | ** |
| S8_52724145 | 8 | 52.72 | 1.67 | NS | 19 | ** | N | 3.28 | NS |
| S8_53308674 | 8 | 53.31 | 0.45 | NS | 10.88 | * | N | 1.28 | NS |
| S8_54355956 | 8 | 54.36 | 1.51 | NS | 11.13 | * | N | 2.86 | NS |
| S8_54678607 | 8 | 54.68 | 1.68 | NS | 12.71 | * | N | 3.04 | NS |
| S8_55352351 | 8 | 55.35 | 1.54 | NS | 12.83 | * | N | 3.12 | NS |
| S9_10225531 | 9 | 10.23 | 9.1 | NS | 30.5 | ** | N | 12.7 | ** |
| S9_11731715 | 9 | 11.73 | 10.18 | * | 12.43 | * | Y | 11.1 | *** |
| S9_11977317 | 9 | 11.98 | 4.15 | NS | 24.5 | *** | N | 6.87 | NS |
| S9_14882885 | 9 | 14.88 | 4.65 | NS | 16 | * | N | 6.81 | NS |
| S9_15158378 | 9 | 15.16 | 2.48 | NS | 11.33 | * | N | 4 | NS |
| S9_31580899 | 9 | 31.58 | 20.33 | *** | 8.6 | NS | N | 13 | *** |
| S9_40134888 | 9 | 40.13 | 3.16 | NS | 30 | *** | N | 5.53 | NS |
| S9_43649628 | 9 | 43.65 | 4.4 | NS | 15.33 | ** | N | 6.52 | NS |
| S9_47539817 | 9 | 47.54 | 15.25 | ** | 32 | *** | Y | 19.8 | *** |
| S9_49554277 | 9 | 49.55 | 14.13 | ** | 17 | ** | Y | 15.2 | *** |
| S9_50198667 | 9 | 50.2 | 7.8 | NS | 13.5 | * | N | 9.43 | ** |
| S9_50719802 | 9 | 50.72 | 6.25 | NS | 11.67 | * | N | 7.73 | NS |
| S9_51718314 | 9 | 51.72 | 4.43 | NS | 28.33 | *** | N | 7.19 | NS |
| S9_55264760 | 9 | 55.26 | 2.33 | NS | 11.13 | * | N | 3.93 | NS |
| S9_55329149 | 9 | 55.33 | 0.81 | NS | 15.8 | ** | N | 1.98 | NS |
| S9_56692240 | 9 | 56.69 | 0.98 | NS | 29 | *** | N | 2.57 | NS |
| S9_57335220 | 9 | 57.34 | 6 | NS | 16 | * | N | 8.35 | * |
| S9_57582717 | 9 | 57.58 | 5.89 | NS | 15.17 | ** | N | 8.12 | * |
| S9_58062014 | 9 | 58.06 | 3.52 | NS | 12.17 | * | N | 5.19 | NS |
| S10_4382269 | 10 | 4.38 | 11.86 | * | 21.33 | ** | Y | 14.7 | *** |
| S10_8603485 | 10 | 8.6 | 15.38 | ** | 6.23 | NS | N | 9.71 | ** |
| S10_8803504 | 10 | 8.8 | 10.63 | * | 20.33 | * | Y | 13.3 | ** |
| S10_23708355 | 10 | 23.71 | 30 | *** | 3.63 | NS | N | 8.22 | * |
| S10_28674405 | 10 | 28.67 | 17.17 | ** | 18 | ** | Y | 17.5 | *** |
| S10_36575703 | 10 | 36.58 | 52 | *** | 9 | NS | N | 17.6 | *** |
