# Supplementary material for: Transmission Genetics of a Sorghum bicolor × S. halepense Backcross Populations
Source: Front Plant Sci. 2020 Apr 30;11:467. doi: 10.3389/fpls.2020.00467 (PMC7203413; doi:10.3389/fpls.2020.00467)
Supplement: FILE S3 — Loci with non-Mendelian segregation combined for two BC1F1 populations of S. bicolor BTx623 × S. halepense G9E. [file Data_Sheet_3.docx]

**Adapter Sequence:**

**FC2, 5-ACACTCTTTCCCTACACGACGCTCTTCCGANNNNNN-3**

**FC1, 3-GTTCGTCTTCTGCCGTATGCTCGAGAAGGCTnnnnnnGC p-5**

**Details for each chromosome:**

For chromosome 1, the total of six H4-derived linkage groups had average segregation ratios of 3.84 (1A), 1.72 (1B), 1.73 (1C), 1.64 (1D), 0.92 (1E) and 0.28 (1F); and four H6-derived groups had 3.82 (1A), 2.72 (1B), 1.48 (1C) and 0.21 (1D), Linkage groups 1B, 1C and 1D segregated with similar ratios and covered different parts of chromosome 1. The two *S. halepense* enriched groups (1A for both populations) and the two *S. bicolor* enriched groups (1F for H4, 1D for H6) both covered nearly all of chromosome 1. No groups with average segregation ratios not significantly different from 1:1 were found covering the short arm of chromosome 1 for the H6-derived population (Table 1, 2 and Figure 2).

For chromosome 2, the total of four H4-derived linkage groups had average segregation ratios of 3.80 (2A), 2.54 (2B), 1.23 (2C) and 0.33 (2D); and three H6-derived groups had 5.67 (2A), 1.63 (2B) and 0.22 (2C). *S. halepense* enriched linkage groups 2A and 2B in the H4-derived population largely covered euchromatic regions of chromosome 2, but absence of markers from possible pericentromeric regions (40-60Mb) makes it difficult to coalesce these two linkage groups (Figure 3). The two *S. halepense* enriched groups (2A and 2B) in H4 and one (2A) in the H6- derived population covered most of chromosome 2, and the two *S. bicolor* enriched groups (2D from H4, 2C from H6) also covered most of chromosome 2. Linkage groups segregating with ratios of 1 (2B and 2C in H4, 2B in H6) are only concentrated on the long arms of chromosome 2, indicating segregation distortion and possible enrichment of *S. halepense* alleles on the short arms.

For chromosome 3, the total of four H4-derived linkage groups had average segregation ratios of 3.51 (3A), 1.52 (3B), 1.02 (3C) and 0.45 (3D); and five H6-derived groups had 3.93 (3A), 3.39 (3B), 1.04 (3C), 0.95 (3D) and 0.26 (3E). The one *S. halepense* enriched group (3A) from the H4-derived population and two (3A and 3B) from the H6-derived population, and the two *S. bicolor* enriched groups (3D from H4 and 3E from H6) both covered most of chromosome 3 with markers concentrated in euchromatic regions. Linkage groups 3C and 3D from the H6-derived population are more concentrated in the central part of chromosome 3 compared to *S. bicolor* and *S. halepense* enriched groups (Figure 2).

For chromosome 4, the total of four H4-derived linkage groups had average segregation ratios of 3.51 (4A), 1.52 (4B), 1.02 (4C) and 0.45 (4D); and four H6-derived groups had 4.33 (4A), 2.64 (4B), 0.58 (4C) and 0.27 (4D). Linkage groups 4B and 4C, each covering different portions of chromosome 4 with similar segregation ratios, might come from the same homologous group. Two *S. halepense* enriched groups (4A for both H4 and H6) and two *S. bicolor* enriched groups (4D for both H4 and H6) largely covered chromosome 4 with markers concentrated in euchromatic regions. No chromosome 4 linkage groups segregating with an average ratio not significantly different from 1:1 were found in the H6-derived population.

For chromosome 5, the total of three H4-derived linkage groups had average segregation ratios of 2.17 (5A), 0.89 (5B) and 0.39 (5C); and three H6-derived groups had 6.56 (5A), 0.70 (5B) and 0.19 (5C). The one *S. halepense* enriched group (5A) in the H4-derived population only covers a small portion of the short arm of chromosome 5, while the one in the H6-derived population covers the long arm (Figure 3). The two *S. bicolor* enriched groups (5C for both H4 and H6) cover most of chromosome 5 with markers mostly in the euchromatic regions. Linkage groups segregating with average ratio of 1 were more concentrated in the middle of the chromosome compared to *S. bicolor* and *S. halepense* enriched groups.

For chromosome 6, the total of three H4-derived linkage groups had average segregation ratios of 2.91 (6A), 1.70 (6B), and 0.42 (5C); and five H6-derived groups had 1.91 (6A), 1.24 (6B), 1.24 (6C), 0.91 (6D) and 0.37 (6E). Linkage groups 6B, 6C and 6D from the H6-derived population, each covering a separate portion of chromosome 6 with similar segregation ratios, might come from the same homologous chromosome. The *S. halepense* enriched groups (6A) only cover 50-60Mb of sorghum chromosome 6 and were only segregating with ratios of 2.91 and 1.9 for the H4 and H6-derived populations respectively, lower than the other linkage groups. An *S. bicolor* enriched group in the H4 population (6C) covered 40-60Mb, while a *S. bicolor* enriched group in the H6 population (6E) covered all of chromosome 6 (Figure 3). Linkage groups segregating with ratios not significantly different from 1 covered the entire physical chromosome.

For chromosome 7, the total of four H4-derived linkage groups had average segregation ratios of 3.34 (7A), 1.15 (7B), 0.97 (7C) and 0.39 (7D), and four H6-derived groups had 5.63 (7A), 1.66 (7B), 1.48 (7C) and 0.47 (7D). Linkage groups 7B and 7C of the H6 population had similar segregation ratios and covered different chromosomal regions, indicating that they might come from the same homologous chromosome. The two *S. halepense* enriched groups (7A for both population) and the two *S. bicolor* enriched groups (7D for both populations) were largely concentrated in the euchromatic regions of sorghum chromosome 7. Linkage groups segregating with average ratios not significantly different from 1 cover most of chromosome 7.

For chromosome 8, the total of four H4-derived linkage groups had average segregation ratios of 3.78 (8A), 1.48 (8B), 1.18 (8C) and 0.27 (8D), and two H6-derived groups had 5.32 (8A) and 0.61 (8B). Both *S. halepense* enriched groups (8A in both populations) and *S. bicolor* enriched groups (8D in H4, 8C in H6) covered most of chromosome 8. Interestingly, linkage groups segregating with average ratios not significantly different from 1 were absent from the H6-derived population and that from the H4 population only covered a small distal portion of chromosome 8.

For chromosome 9, the total of two H4-derived linkage groups had average segregation ratios of 4.40 (9A) and 0.36 (9B); and three H6-derived groups had 4.82 (9A), 1.32 (9B) and 0.27 (9C). The two *S. halepense* enriched groups (9A in both populations) covered chromosome 9 with more markers on its long arm, while two *S. bicolor* enriched groups largely covered chromosome 9 with most markers on the euchromatic regions. No linkage group segregated with average ratio not significantly different from 1 in the H4-derived population while in H6, the only one (9B) was concentrated on the long arm of chromosome 9.

For chromosome 10, the total of four H4-derived linkage groups had average segregation ratios of 3.83 (10A), 1.38 (10B), 0.94 (10C) and 0.32 (10D); and three H6-derived groups had 3.14 (10A), 1.45 (10B) and 0.33 (10C). Both *S. halepense* enriched groups (10A in both populations) and *S. bicolor* enriched groups (10D and 10C in H4 and H6-derived populations, respectively) covered chromosome 10 with markers mostly in the euchromatic regions. The marker distribution patterns of linkage groups segregating with an average ratio not significantly different from 1 is similar to that of *S. halepense* and *S. bicolor* enriched groups.
